# Supplementary material for: Effects of Dietary Glucose Oxidase Supplementation on the Performance, Apparent Ileal Amino Acids Digestibility, and Ileal Microbiota of Broiler Chickens
Source: Animals (Basel). 2021 Oct 8;11(10):2909. doi: 10.3390/ani11102909 (PMC8532941; doi:10.3390/ani11102909)
Supplement: Supplementary file 1 [file animals-11-02909-s001.zip › animals-1369992-supplementary.pdf]

Table S1. Effect of dietary glucose oxidase addition on the carcass performance of broilers at 42 days of age

Treatments[1]	Carcass yield[2] (%)	Eviscerated carcass yield[2] (%)	Thigh yield[3] (%)	Breast yield[3] (%)	Abdominal fat weight[3] (%)
Control	89.91	72.37	28.76	21.61	1.41
E250	91.09	74.33	27.50	21.16	1.34
E500	90.50	72.63	29.32	20.22	1.48
E1000	90.67	74.16	28.95	21.66	1.44
SEM	0.94	1.30	0.80	0.57	0.11
p-value	0.851	0.482	0.374	0.190	0.846

[1] Control, E250, E500, and E1000 represented the basal diet supplemented with 0, 250, 500, and 1000 U glucose oxidase/kg diet, respectively.

[2] Calculated as a percentage of live bodyweight.

[3] Calculated as a percentage of eviscerated carcass weight.

Table S2. *p* values obtained from the comparison of microbiol unweighted UniFrac (gray background) or weighted UniFrac (white background) distance using the PERMANOVA tests.

Treatments[1]	Control	E250	E500	E1000
Control		0.196	0.738	0.300
E250	0.049		0.033	0.164
E500	0.266	0.012		0.115
E1000	0.074	0.294	0.042	

[1] Control, E250, E500, and E1000 represented the basal diet supplemented with 0, 250, 500, and 1000 U glucose oxidase/kg diet, respectively.

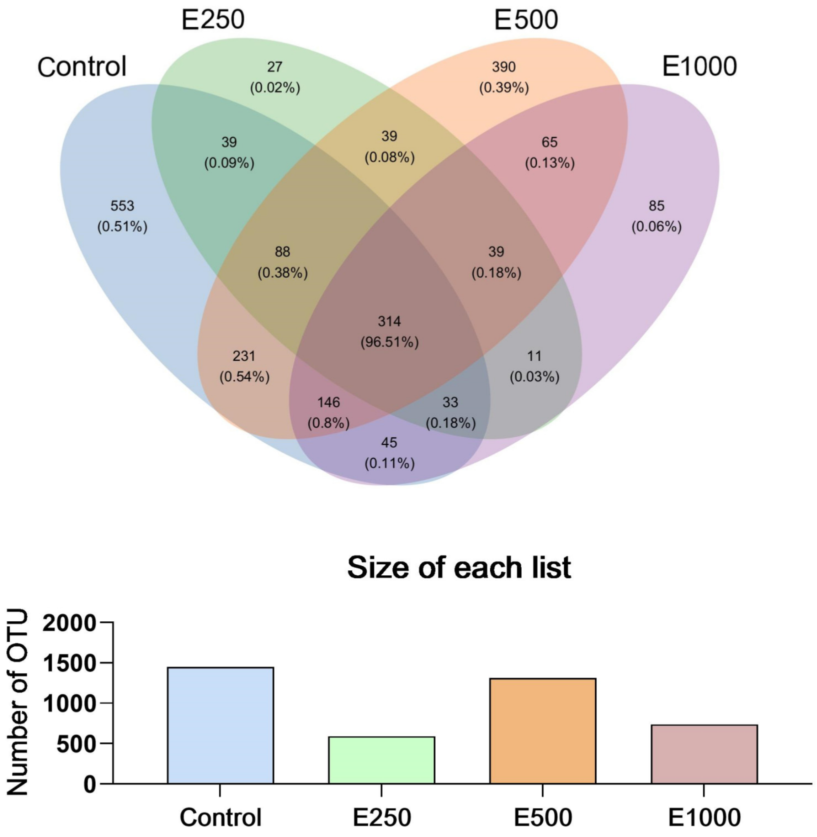

Figure S1. Effects of dietary glucose oxidase addition on the composition of bacterial OTUs in the ileal microbiota of broiler chickens. Venn diagram showing the occurrence of bacterial OTUs identified in 16S rRNA fragment sequencing of ileal microbiota of chickens fed a basal diet (Control) or the basal diet supplemented with 250 (E250), 500 (E500), or 1000 U glucose oxidase/kg diet.
